# Abdominal Trauma: Never Underestimate It

**DOI:** 10.1155/2011/850625

**Published:** 2011-10-12

**Authors:** Aakash N. Bodhit, Anjali Bhagra, Latha Ganti Stead

**Affiliations:** ^1^Department of Emergency Medicine, University of Florida College of Medicine, Gainesville, FL 32610, USA; ^2^Department of Internal Medicine, Mayo Clinic, Rochester, MN 55905, USA

## Abstract

*Introduction*. We present a case of a sports injury. The initial presentation and clinical examination belied serious intra-abdominal injuries. *Case Presentation*. A 16-year-old male patient came to emergency department after a sports-related blunt abdominal injury. Though on clinical examination the injury did not seem to be serious, FAST revealed an obscured splenorenal window. The CT scan revealed a large left renal laceration and a splenic laceration that were managed with Cook coil embolization. Patient remained tachycardic though and had to undergo splenectomy, left nephrectomy, and a repair of left diaphragmatic rent. Patient had no complication and had normal renal function at 6-month followup. *Conclusion*. The case report indicates that management of blunt intra-abdominal injury is complicated and there is a role for minimally invasive procedures in management of certain patients. A great deal of caution is required in monitoring these patients, and surgical intervention is inevitable in deteriorating patients.

## 1. Introduction

Abdominal trauma, especially those caused by blunt force is a leading cause of morbidity and mortality in all age groups, but it is one of the most challenging conditions emergency department physicians encounter because of varied presentations [1]. The difference in severity between presenting symptoms and actual injuries in a significant number of cases makes the rapid diagnosis and management for such patients more complex.

While managing abdominal trauma patients, it should be kept in mind that a seemingly minor injury can also be a cause for major intra-abdominal organ injuries, and rapid yet efficient detection of such injuries should be the goal to significantly improve the patient outcomes.

## 2. Case Presentation

A 16-year-old baseball player presented to our emergency department after trauma during a game. Patient was playing baseball when he ran into another player injuring his left chest and left upper abdomen. Subsequently, he lost consciousness and on regaining consciousness, he was diaphoretic and confused and complained of left chest and upper abdominal pain. Patient was flown from the scene on a backboard with C collar immobilization in place. In the ED, the patient complained of back and abdominal pain. On exam, the patient was alert and oriented with stable vitals. There was marked tenderness to palpation in left lower back and over the lumbar spine. Chest radiograph was negative. FAST scan revealed a clear Morrison's pouch; however, the splenorenal window was obscured. Urgent CT of the chest and abdomen was obtained to confirm this positive FAST exam finding.

**Figure 1:**
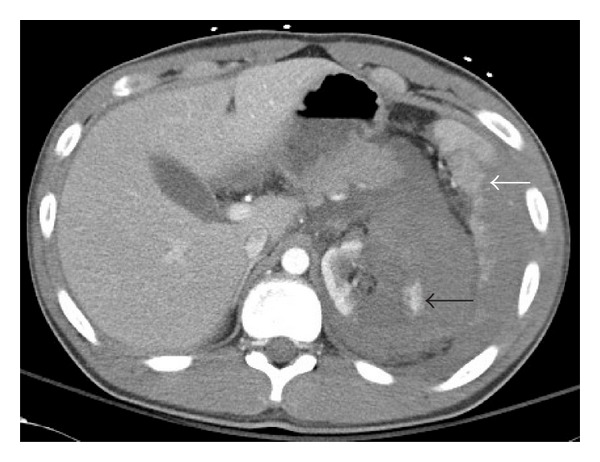
Axial CT of the abdomen revealing extensive left renal hematoma and laceration (white arrow). Also note the splenic laceration with subcapsular hematoma (black arrow).

**Figure 2:**
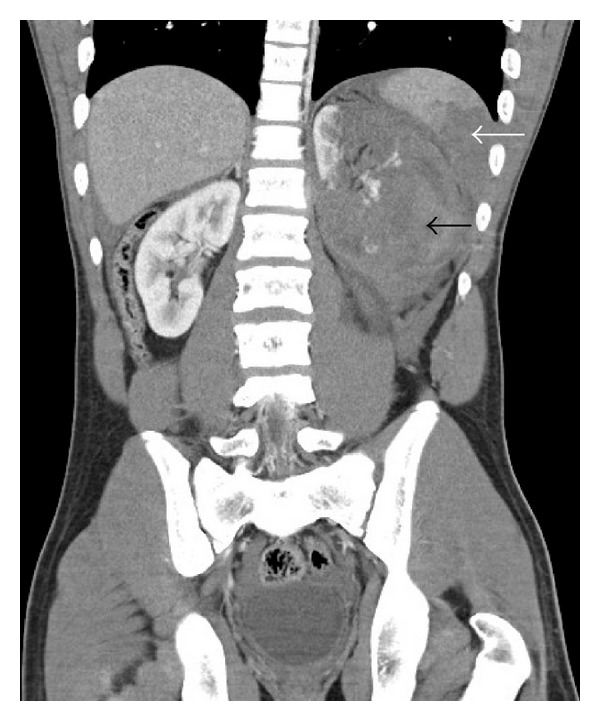
Coronal reconstruction revealing extensive left renal (black arrow) and splenic injuries (white arrow).

CT scan revealed a large left renal laceration extending into the renal hilum with associated hematoma measuring approximately 12 cm (white arrow, Figures 1 and 2), tracking down the left retroperitoneum into the pelvis with evidence of extravasation. A large splenic laceration with moderate sized subcapsular hematoma (black arrow, Figures 1 and 2) was also noted. The lower left three ribs were fractured. The patient underwent urgent Cook coil embolization of both viscera. Subsequently, due to persistent tachycardia, he underwent a laparotomy which revealed venous blood oozing from both the injured viscera with left diaphragmatic rent and hemothorax. He underwent splenectomy, left nephrectomy, and diaphragmatic rent repair with no postoperative complications. On six-month followup, he had normal kidney function. 

## 3. Discussion

Abdominal trauma is a very common presentation in emergency department patients, and early detection of intra-abdominal injuries can be challenging in some patients, particularly those with seemingly minor trauma or normal clinical examination [1]. In such patients, noninvasive diagnostic techniques such as focused ultrasonography and CT scan can help diagnose intra-abdominal organ injuries [2, 3]. It should be noted that though FAST is an important tool for the diagnosis of abdominal organ injuries in trauma patients, it has shortcomings such as relatively low sensitivity as compared to CT scan [4]. 

Nonoperative management of hemodynamically stable patients is better in patients with solid organ injuries due to abdominal trauma that are admitted in a facility where tertiary level health care is available round the clock [5]. It is necessary to monitor such patients with great caution to detect deterioration of health readily. There is a certain role for angioembolization in these patients, but it needs to be decided on individual patient's characteristics. Patients with deteriorating clinical signs when they are being managed nonoperatively should go to operating room for laparotomy [6].

## 4. Conclusion

An abdominal blunt injury which does not seem serious on initial presentation and on clinical examination can actually be a serious intra-abdominal organ injury. Management of this subtype of patients requires a cautious approach that includes a possible role of noninvasive or minimally invasive methods, but any deterioration of clinical signs and symptoms should be followed by surgical management.
